# Evaluating the Viability of Virtual Reality for Children’s Food Choice Research: Comparative Mixed Methods Study

**DOI:** 10.2196/88476

**Published:** 2026-07-02

**Authors:** Deepti Aggarwal, Thuong Hoang, Sze-Yen Tan, Mohammadreza Mohebbi, Catherine G Russell

**Affiliations:** 1 School of Information Technology, Faculty of Science Engineering and Built Environment, Deakin University, Geelong, 3125, Australia, 61 92445280 ext 03; 2 School of Exercise and Nutrition Sciences, Faculty of Health, Deakin University, Geelong, Australia; 3 Faculty of Health, Biostatistics Unit, Deakin University, Geelong, Australia

**Keywords:** virtual reality, portion size, food choices, children, embodiment, immersion

## Abstract

**Background:**

Virtual reality (VR) systems offer promising potential as a controlled platform to investigate human behaviors specifically related to food choices. Yet, little is known about its viability for conducting food choice studies with children, thus limiting the development of public health research.

**Objective:**

This study aimed to investigate the viability of VR technology for understanding children’s food choices, focusing specifically on perceptual differences between VR and real-life (RL) settings. We examined how children perceive and interact with food portion sizes and container sizes in VR as compared with an equivalent RL scenario.

**Methods:**

A within-subject, mixed methods study was conducted with 437 children aged 5‐12 years at a science museum. Participants engaged in a standardized food selection task for a simulated breakfast scenario, choosing portions of cereal and milk in 2 conditions: a head-mounted VR environment and a corresponding RL physical setup. Children’s food selection behaviors were quantitatively compared across 3 independent variables: condition (VR vs RL), food healthiness (healthy vs unhealthy options), and container size (small, medium, and large). Qualitative and quantitative data were collected via postsession questionnaires assessing presence, embodiment, and simulator sickness, alongside detailed interaction logs from the VR environment. Data analysis used statistical comparisons and thematic analysis.

**Results:**

The findings revealed both behavioral consistency and significant perceptual differences between the VR and RL conditions. A behavioral similarity was identified, as children served significantly larger portions of unhealthy food compared with healthy food in both conditions. Crucially, a difference was observed in size perception: children struggled to accurately match the size of bowls and glasses between the VR and RL conditions. Furthermore, while children reported low feelings of presence and embodiment within the VR scenario, they demonstrated a high degree of control and engagement in the virtual task.

**Conclusions:**

Our findings suggest that current state-of-the-art VR technology presents limitations in its viability for conducting food choice studies with children, particularly concerning accurate size and volume perception. Based on the findings, we provide 4 practical recommendations to guide the future development of immersive food environments, thereby supporting more reliable and ecologically valid food choice research with young populations.

## Introduction

### Background

Within the field of human-computer interaction (HCI), there is a growing interest in designing for and around food practices [1-3], underlined by research in dietary-monitoring systems [4,5], technologies for supporting rich conversations during mealtimes [6,7], and awareness about our food ecosystems such as food waste and carbon emission [8]. Recent works further blend food research with interactive experiences, such as combining real-life (RL) eating with virtual reality (VR) gaming experiences [9], encouraging healthy eating practices through tangible systems [10,11], and creating novel food experiences using emerging technologies such as 3D printers [12]. Recently, Covaci and colleagues [13] presented multiple design illustrations, through several co-design workshops, speculating the design of VR-based food experiences for the everyday context. As such, existing research collectively suggests that it is feasible to enhance eating experiences with interactive technologies for different contexts. In this work, we take a step further and explore the viability of using VR technology as a tool to conduct food choice studies. Unlike existing works that focus on the adult population, we investigate the use of VR technology for younger children (aged 5‐12 years).

On the other hand, existing literature in the nutrition science domain suggests the viability of using VR systems to conduct food choice studies, due to its capability to simulate realistic environments and food-related cues [14-18]. For instance, studies with adults using VR have highlighted key insights into their personal eating practices and the ways they feed their children [14,15,19]. There is, however, a notable gap in understanding the firsthand experience of children about their food choices. This is a crucial area to explore, as a rich understanding of how children make food choices can guide the development of effective programs that promote positive eating practices from an early age [20,21]. Given the rising rates of childhood obesity in many countries [22,23] and the serious health risks it poses later in life [20,22], this research addresses a timely and important topic.

Engaging children in research can, however, be challenging, as the scientific tasks may seem repetitive or boring to them [24]. Researchers have emphasized the need to employ developmentally appropriate procedures and measures for robust data collection [25] as well as to modernize study procedures to increase participation of children in research [26,27]. VR systems offer an engaging environment for children to learn and explore, and their popularity among children and teenagers is increasing [28,29]. A recent survey suggests that 1 in 5 families with children younger than 17 years has a VR headset [30] and that around 70% of 2‐ to 15-year-old children in the United States and United Kingdom reported being fairly or extremely interested in VR [29]. Previous works have investigated the potential of VR to help children deal with painful clinical procedures [31,32], as well as to teach them a wide variety of topics in school settings such as learning to cook [33], Swedish language [34], and science simulations [35]. This research further extends the applications of VR with children by investigating how children interact with simulated food environments in VR and how their interactions differ from RL settings.

**Figure 1.**
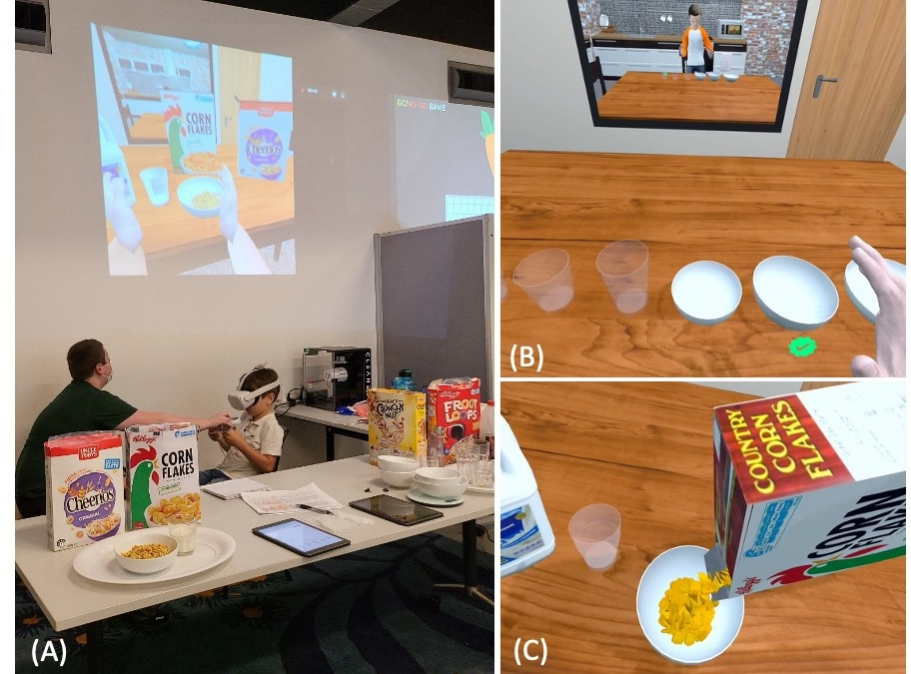
A snapshot of the virtual reality (VR) study booth at the science museum. (A) Children were tasked to make food choice decisions for breakfast items in both VR and real-life scenarios. (B) Participant’s viewpoint in VR during bowl size selection task. (C) Participant’s viewpoint while pouring cereal into a virtual bowl.

This paper investigates the viability of using VR technology to understand children’s food choices. We investigated how children perceive food portion sizes and container sizes in VR and how these perceptions differ from RL scenarios. We specifically focused on only 2 tasks—food portioning (ie, how much can the individual serve themselves) and container selection (ie, which container the individual should select)—as these are the basic tasks required to conduct food choice studies. We conducted a controlled trial with 437 children aged 5-12 years at a science museum (as shown in Figure 1). Not all participants completed the same set of tasks; hence, the sample size for each research question (RQ) varied. Employing a within-subjects study design and mixed methods data collection approach, we present a comparative account of participants’ interactions in a virtual food environment (ie, a virtual kitchen) with an RL museum setting.

This work makes the following contributions: First, our study investigates the firsthand experience of children in making food decisions related to food portion sizes and container sizes. Our findings showed that children served larger portions of unhealthy food than healthy food in both the VR and RL conditions. Second, we discovered that children struggled to accurately match the size of bowls and glasses between the VR and RL conditions. Both findings raise concerns on the viability of using VR technology for food choice studies. Moreover, our study also revealed that children found the VR experience so engaging and fun that it could potentially compromise the scientific rigor needed for data collection. In response, we present 4 recommendations to guide future development of playful VR experiences for children that can still maintain high scientific standards and data quality. Finally, this work extends HCI practices into nutrition science by exploring a novel application of VR technology as a research tool for conducting food choice studies. By introducing methods for testing concepts such as food portion sizes, container size mapping, and health factors related to food items, we have opened new avenues for HCI researchers and nutrition scientists to create robust and impactful VR systems in the future.

### Prior Work

We discuss the related literature around 3 topics: VR as a tool for food research, VR as an engagement tool for children, and children's embodiment in VR.

#### VR as a Tool for Food Research

Researchers in health and nutrition are just beginning to explore the use of VR technology to study eating habits and food choices [14,16]. This field is still new [36], but initial studies have already shown that VR can create realistic food environments that produce convergent results with RL settings [14,36-38]. In food research, participants often perform the same tasks in VR and real-world settings, allowing researchers to directly compare their choices. These studies use a range of virtual environments, such as virtual buffets [14,15], cafeterias or food courts [39,40], and supermarkets [17,37,38,41], to offer participants a wide range of food options. On the other hand, VR systems also allowed researchers to easily change factors such as food color and texture, food venues (bar or restaurant), and social settings, as per the study requirements. It also provided a way to collect objective data related to food selection such as the order in which items are chosen, portion sizes of different food items, and the total weight of the selected food.

As such, prior research suggests the viability of using VR to conduct food studies [14-18]. Researchers have argued that the realism offered by VR technology addresses the existing issues with traditional measures to record personal food choices [25,42,43]. For instance, assessing food choices through real food is challenging due to the associated high cost of buying real food while also managing food safety (such as food handling) and food preferences (eg, allergies, vegan or vegetarian, etc). Limiting food choices to overcome the cost issues is not a viable solution as it can impact the generalizability of the study findings [43]. On the other hand, conducting studies with fake foods is another potential solution, which allows researchers to include a wide variety of food items for greater experimental control. However, fake foods lack in simulating realistic environments that significantly influence the food choices, hence limiting generalizability and ecological validity of the study findings [42].

To understand the food practices of children, several studies have employed proxy measures and investigated parents’ feeding practices [14-16]. Persky and colleagues [15] compared parents’ pasta selection for their children in VR versus RL conditions and reported a high correlation. Parents in the study also felt that the VR environment was realistic and that their virtual food choices accurately reflected their RL habits, both in terms of portion size and healthiness. Similarly, another study [44,45] used a VR buffet to raise awareness about childhood obesity among mothers whose children were genetically vulnerable to being obese in childhood and later. The study was conducted with overweight and obese mothers, where they were required to select food they believed their child would eat through pointing-based interaction in VR. The researchers observed the mothers’ decisions and measured the portion sizes they chose and accordingly educated them on appropriate food portion sizes for their children.

There is a limited understanding of the firsthand experiences of children’s food selection in a virtual food environment, which this research aims to address. Our work builds upon the recent HCI research [13] that has explored the potential applications of VR for food-related environments. The authors described 18 themes to develop “food metaverse” that can help people connect with food and adopt sustainable practices. To design similar food metaverse experiences for children, it is important to understand their perceptions of food portion sizes and container size mapping, as these are fundamental tasks in any food choice study. Furthermore, by comparing how children perceive and interact with food in both VR and RL, we can determine whether VR is a valid tool for studying this age group. Our goal is to address these gaps by providing a detailed comparison of children’s interactions in a virtual food environment versus an RL setting, focusing on portion sizes and container size estimation.

#### VR as an Engagement Tool for Children

VR has mainly been used as an educational tool to engage children in topics that are otherwise complex to teach in real-world settings. For instance, Gorman and colleagues [33] used VR with 360-degree images and videos to teach middle schoolers about food hygiene and safe cooking practices. The VR setup successfully overcame challenges related to the shortage of cooking facilities and teaching staff and created an engaging classroom where children were highly motivated to learn. In another study, researchers [46] designed a virtual kitchen where 7‐ to 9-year-old children could prepare their breakfast and get relevant feedback on their food choices. The study revealed that VR offered a promising environment, as children enjoyed learning about the nutritional value of various breakfast items. Another work [45] used a VR supermarket to teach 6- to 13-year-olds about the environmental impact of their food choices. The study found that all participants could recall the information presented in text and images format, but only those children older than 10 years were able to fully understand the messages. Interestingly, those who understood the information reported feeling sorry about the negative environmental impact of their food habits and expressed a desire to change their habits for the better. We extend the applications of VR for children beyond education and investigate their interactions with food and containers in a food choice study.

#### Children’s Interactions in VR

A significant amount of works have explored how adults and older adults embody themselves in VR environments for different contexts, such as for physiotherapy training [47], combating social isolation in aged care facilities [48], and for gaming purposes [9]. However, there is a notable gap in understanding how children interact and embody themselves in VR (as also emphasized by a recent review paper) [49]. Among the few studies, Keenaghan and colleagues [50] compared how adults and 5-year-old children perceive different-sized avatars and objects in VR. Participants were asked to estimate sizes of their body and objects in a virtual “tea party” environment. The virtual avatar was manipulated relative to the participant’s actual height. The study revealed that the reported degrees of embodiment across adults and children were comparable. Participants’ perception of the virtual objects was influenced by their virtual body size, that is, they felt that the virtual objects were bigger when they embodied a smaller body and vice versa in a larger body. The notable finding was that unlike adults, children also felt that their (actual) body size increased in the large body condition; however, they did not report getting smaller in the small body size condition. The authors hypothesized that since children are more familiar with their growing body, they are more likely to notice a change in body size than adults. Also, children mainly reported feeling bigger and not smaller because shrinking of bodies is not a natural phenomenon. Previous research also suggests that children are more sensitive to their own-body representation as they must keep track of a constantly changing and growing body along with other sensory developments [51].

Weijs and colleagues [52] similarly compared a full-body embodiment of adults and 8- to 12-year-old children. The authors investigated participants’ responses to synchrony of the avatar’s movements (ie, avatar moving with the same or different speed as that of the participants) and appearance of the avatar as human and skeleton. Their study highlighted that both children and adults showed highest ownership for the virtual avatar for the synchronous condition, and that the ownership reduced with increased asynchrony. Also, higher ownership and agency were reported for human avatars than the skeleton avatars by children and adults. The notable difference was that children demonstrated a stronger sense of agency than adults, both with increasing temporal mismatches and with skeleton avatars. The difference in agency (ie, the sense of being in control) suggests the developmental differences in children and adults. Prior research [53] revealed that children aged up to 8 years move from relying solely on visual information to integrating multiple senses to develop a better sense of their own bodies.

In a recent study [54], researchers compared children’s perception of human, animal, and anthropomorphized creatures in VR and revealed that the animal avatars received the most positive descriptions from children aged 5-9 years. Since animal creatures are farther away from human-likeness, realistic behaviors were not expected from them. On the other hand, human and anthropomorphized creatures were expected to have highly realistic behaviors. The virtual character’s movements, blinking of eyes, and facial features such as head size or hair color were found strange and unreal by children. Similarly, another study [55] investigated the perception of 6- to 8-year-old children related to presence and realism in VR. Through a qualitative study of the VR experience where children swam in a pool with dinosaurs, the study highlighted how VR challenged children’s understanding of reality when dinosaurs were treated as real. Children also tested the reality of VR by doing certain activities such as holding breaths and comparing the number of fish the dinosaurs consumed in VR as opposed to their actual diet. While some children verbally described the VR experience as fake, they still reported physiological and emotional responses to VR exposure. The study concluded that although children knew that dinosaurs were extinct, VR was still able to create alternate perceptions of what is real for them. We take inspiration from these works and further extend the literature on VR by investigating children’s perceptions of food and containers in a food choice study. Next, we describe our study design.

## Methods

### Ethical Considerations

The project was approved by the Deakin University ethics committee (ethics number 2019‐393), including approval for the use of commercial VR headsets with children younger than the minimum recommended age of 13 years. Consent from parents and assent from children were obtained prior to the study. Once consent was obtained, parents provided the demographic details of the participants at the entry point through a questionnaire. The study was conducted exclusively with child participants. Upon recruitment at the study's entry point, each participant was assigned a unique alphanumeric ID code. This ID was utilized across all data collection platforms—including VR system logs and Qualtrics survey responses—to ensure seamless data integration. All subsequent statistical analyses were conducted using these IDs to maintain participant confidentiality.

### Study Design

Our study was a part of a larger children’s eating and weight project, which was conducted at a science museum in a metropolitan city in Australia. The museum is popular for featuring interactive installations and exhibitions related to science and technology and aims to expose children to innovation and its societal impact. The project was set up with multiple activities within a dedicated room within the museum, with a single entry and exit point. Our study was one of the activities within the room. Interested families approached the room at the museum, where banners were placed to invite participants. The volunteers introduced the families to the project and the associated activities. All participants, including children and parents, were recruited at the entrance. Parents were informed that VR activity may cause motion sickness, so children with motion sickness history should not participate. However, no parent mentioned any motion sickness history for their children.

To mitigate the risk for this age group, we ensured that the participants used the VR headset in a seated position. Additionally, to help prevent simulation sickness in children, we limited the duration of the VR experience to less than 5 minutes. We used Oculus Quest 2, as the study occurred before the rebranding of Oculus Quest 2 to Meta Quest 2. The study was conducted over 5 consecutive weekends (Saturdays and Sundays) for a total of 10 days over a school holiday period. In total, 437 children aged 5‐12 (mean 8.2, SD 2.0) years participated in the study. Out of the 437 participants, 175 were female and 190 were male. Gender information for the remaining 72 participants was unavailable, which was attributed to either unstable internet connection at the museum or incomplete demographic information provided by the parents. Since the study was conducted at the museum, we had no control over the gender distribution and allowed all eligible participants to participate in the study. Participants did not receive any monetary compensation for their participation. We also noted that children younger than 16 years had free access to the museum; however, the accompanying parents were required to buy a ticket.

### Research Questions

Building upon existing works that highlight the potential of VR technology for conducting food choice research [15,18,46], we designed a VR experience to investigate children’s interactions in the virtual food environment. The main RQ we explored in our study was *what factors affect the use of VR as a medium for food choice research with children?* We compared the participants’ interactions within a simulated food environment in VR with that of RL setting at the museum.

The main RQ is broken down into different aspects.

1. RQ1: How does VR influence the measurement of key variables in food choice studies (food portion size, container size, and additional characteristics of healthy and unhealthy)?
2. RQ2: How does the children’s experience (presence, embodiment, simulator sickness, and repeatability) of being in VR affect its usage as a medium for food choice research?
3. RQ3: How do children’s demographic characteristics (gender and age) affect the VR usage as a medium for food choice research?

### Study Parameters

**Figure 2.**
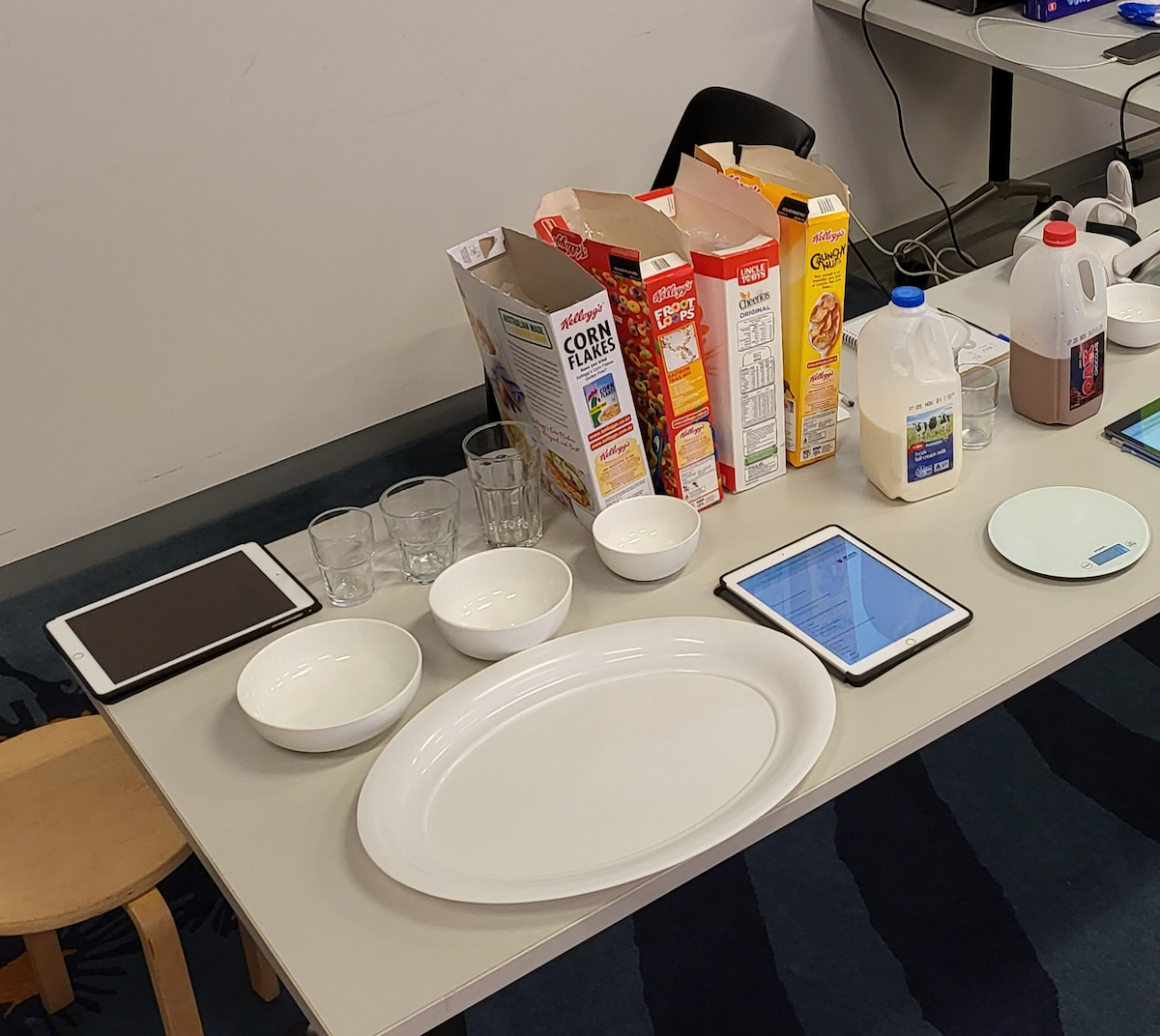
A snapshot of the study station for the real-life condition: 3 sizes of bowls and glasses (small, medium, and large), a large plate to prevent milk spillage, a tablet to fill out the questionnaire, and a scale to weigh the results afterward.

The study was designed around the everyday activity of breakfast, as this is when children are most likely to serve themselves food. Participants were asked to imagine that it was breakfast time, and they needed to serve themselves cereal and milk. The 2 main conditions for the study were VR and RL. The setting for the VR condition was a virtual kitchen environment, and the setting for the RL condition was the museum. The independent variables were (1) food healthiness with 2 levels: healthy and unhealthy, and (2) container size with 3 levels: small, medium, and large. Table 1 provides a summary of the study design. These 2 independent variables were applied to 2 food options: cereals and milk. Figure 2 provides a snapshot of the study station.

**Table 1.** Summary of our 2×2×3 study design. Food options included breakfast cereal and milk, and container options included bowl and milk.

Independent variable	Levels
Condition	Real life and virtual reality
Food healthiness	Healthy and unhealthy
Container size	Small, medium, and large

Our choice of cereal and milk was guided by their health star rating and sugar content information available on the package: health star rating is a standard method to define the healthiness of a product in our country [56,57]. A large amount of sugar is not desirable in young children, as sugar intake has been associated with potentially adverse consequences, such as dental caries, obesity, and coronary heart diseases, in the long run [58]. Two healthy cereals in the study included Uncle Toby’s Cheerios (Health Star Rating of 4 [59] and sugar content as 14.6 g/100 g) and Kellogg’s corn flakes (Health Star Rating of 3.5 and sugar content as 10 g/100 g). Two unhealthy cereals included Kellogg’s Froot Loops (Health Star Rating of 2 and sugar content as 38.8 g/100 g) and Kellogg’s crunchy nuts (Health Star Rating of 2 and sugar content as 35 g/100 g).

For cereals, we also maintained the consistency in shape to avoid any other potential influence on the findings [60]. For example, using dinosaur-shaped cereal can influence children’s choice as opposed to using flat-shaped cereals (ibid.). Hence, we selected breakfast cereals of similar shapes for both healthy and unhealthy options. For instance, Uncle Toby’s Cheerios (healthy option) and Kellogg’s Froot Loops (unhealthy option) both are ring-shaped. Similarly, Kellogg’s corn flakes (healthy option) and Kellogg’s crunchy nuts (unhealthy option) both are flat.

On the other hand, options for milk included plain milk as a healthy option (with sugar content as 4 g/100 g) and chocolate milk as unhealthy (with sugar content as 10.6 g/100 g). For cereal bowls and milk glasses as containers, we purchased crockery sets (bowls and glasses) with 3 sizes: small, medium, and large from the local department store. Participants were instructed not to consume any food during the RL trial to prevent potential risks related to food allergies and food hygiene.

In the study, we did not ask nor expect participants to distinguish between healthy and unhealthy foods. Instead, the research team randomly assigned children with different food options. The healthiness variable was used mainly to investigate food portion size in RQ1. Consequently, no visual labels were added on the boxes to categorize them as healthy or unhealthy. In previous research, factors such as taste, health, and social settings have all been shown to affect how much food people serve themselves [3,61]. While we did not investigate taste or social influence in our study, we specifically investigated how the health factor impacts food portion size. Some studies suggest that foods with high sugar content are often perceived as tastier than those with less sugar [62], thus making the health-taste-portion size connection relevant for our study.

In the RL condition, products were replenished as needed to maintain consistency across participants. To preserve the structural integrity of the cereal, items were never handled directly; instead, they were poured into and out of the containers, preventing physical breakdown. In contrast, milk was replenished on a daily basis due to its shorter shelf life.

Due to the challenges of letting children handle real food in a public museum, our study adopted a different approach for the RL and VR conditions (more details under “Methodological Challenges” section). In the VR condition, children served the food themselves. In the RL condition, however, a researcher served the food based on the child’s verbal instructions. We made this choice to maintain a level of agency for the child in both conditions. This difference, particularly in how the stop pouring” action was executed (verbally in RL vs by hand movements in VR), does create a confounding variable. However, we expect that it will not impact our results, as RQ1 aims to investigate children’s decision-making process about food portion sizes as a cognitive task and not as a motor skill task of serving food. Therefore, we expect the final portion sizes to be comparable in both conditions.

### VR Experience

We designed a virtual environment of a typical home kitchen with a range of food products, crockery, and cutlery along with other typical everyday objects (eg, vase and microwave) to create a realistic environment (Figure 1 provides a summary of the study). Participants appeared as an avatar seated at a kitchen table, with virtual cereal boxes and milk cans within their reach. We used the same cereal and milk brands for both the VR and RL conditions. A virtual bowl and glass were placed in front of the participants on the kitchen table. Participants were required to press the controller buttons to grab an object (bowl, glass, cereal box, and milk can) from the table and tilt the controller to pour cereals and milk into the containers (Figure 1C). We placed a virtual mirror in front of the participant, on the other side of the kitchen table, to induce ownership of the self-avatars [63] (Figure 1B). Using Unity meter units, virtual bowls and glasses were matched with the dimensions of store-bought physical containers for all sizes: small, medium, and large (default scale of 1 unit equals 1 meter).

To calculate the cereal weight in VR, we applied a particle system that provided an estimate weight of each cereal type. We measured the average weight of cereal for each of the 4 types, performed a sample test by measuring the real weight of full and half-full bowls for all sizes, and finally calibrated the measurement values in VR. To calculate the volume of milk, we applied the rate of pouring based on the tilt angle of the milk container to increase the visual content within the virtual glass. We measured the capacity of the physical glasses for full and half-filled and adjusted the visualization of the virtual glasses to match accordingly. The weight of the RL containers (bowls and glasses) was measured prior to the study.

### Study Procedure

Before starting the study, we conducted a pilot study with 10 children (4 boys and 6 girls; mean age 8.1, SD 1.9 years) recruited through the researchers’ network. The pilot study was conducted 4 weeks before the final study in a VR laboratory at the university campus to check whether the study tasks were age appropriate and whether the children were able to finish the entire study within 10 minutes. Given that the study took place in a crowded science museum, it was essential for each child to complete the tasks quickly to manage the flow of participants within the large room with multiple study stations. The pilot study confirmed the risk of food spillage as some of the younger pilot participants could not comfortably handle the milk can. Thus, we adopted the procedure for a researcher pouring the milk in the RL condition instead of the children. On the other hand, the data collection questionnaire was revised to minimize the time spent at the VR station. Specifically, we simplified the Likert scales by reducing the number of response categories and reduced the number of questions to ensure that they were developmentally appropriate and easily interpretable for children aged 5‐12 years (more about questionnaires is discussed under “Data Collection” section).

We employed a within-subjects study design, where all participants were asked to complete the main task of making the breakfast cereal in 2 conditions: VR and RL. For the first condition, participants were given the following instructions:

*Imagine that you are in your kitchen. It’s a normal school day, and you are preparing breakfast for yourself before you head to school. You will not get to eat again for another 3 hours or so, so prepare yourself a sufficient breakfast. Serve yourself a breakfast cereal and pour yourself a glass of milk to drink.*

Participants were asked to pour milk and cereal into separate containers, so that we could accurately measure the portion sizes of each item individually. For the second condition, participants received the same instruction to serve the portion size that they would like to eat or drink, with the assumption that it would not have changed from the first condition. The first condition and the second condition were alternated between VR first–RL second and RL first–VR second.

For the RL condition, participants were asked to sit on the provided chair, accompanied by a researcher. Each participant was assigned to either the healthy or unhealthy food option. The order was alternated to ensure a balanced number of participants in both groups. A bowl and a glass were placed directly in front of them. Participants did not handle the food directly in RL; rather, the researcher poured the food, following their instructions. The researcher first provided the task instructions to the participants and then picked up the selected cereal and started pouring it into the bowl. Participants were asked to say “Stop” when the desired amount was reached. A similar process was repeated for the drink. The researcher slowly poured the food and constantly checked with the children whether the amount was appropriate. After that, the bowl and glass with the food were weighed on a table scale and the data were recorded in Qualtrics. The actual weight of the corresponding container was later subtracted to retain the weight of the poured food content. Milk was measured by weight rather than volume for consistency. All food contents were poured back into their respective containers for reuse. Figure 2 shows the arrangement of the study station for the RL condition.

For the VR condition, participants were first asked to sit comfortably in the provided chair. A researcher assisted the participants in wearing the VR headset (Oculus Quest 2) and 2 handheld controllers. The interpupillary distance of the headset was adjusted for each participant for the best visual clarity, but the adjustment values were not recorded as data. The researcher ensured that the participant remained seated throughout the session for safety purposes, with a protocol in place to stop the session immediately if a child experienced any VR-related discomfort. However, no adverse effects were observed, and all participants successfully finished the session. The researcher explained to the participants how to operate the handheld controllers and interact with the virtual environment. A practice task was completed before starting the study tasks to avoid novelty. In the virtual kitchen, participants were represented as a virtual avatar seated against a table, with gender and skin tone matched according to the researcher’s observations. The researcher confirmed whether the participant agreed to the chosen gender and skin tone of the virtual avatar before starting the study procedures.

**Figure 3.**
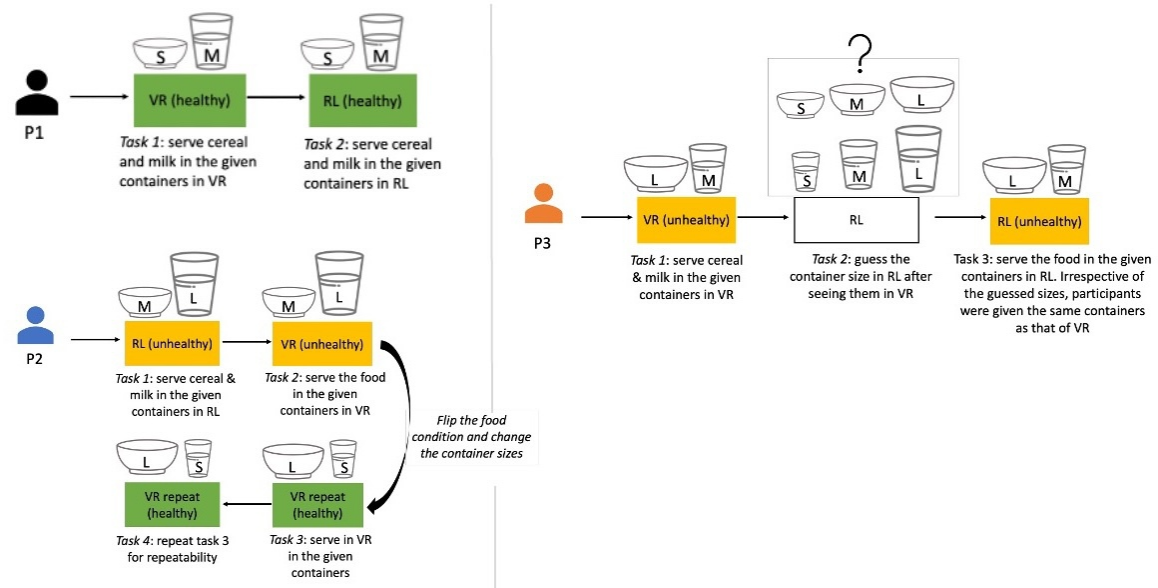
Sample order of tasks for 3 participants completing VR and RL conditions. P1 completed only the main task related to RQ1 portion size for healthy condition. P2 first performed the portion size task and then completed additional tasks to serve the same amount of food in VR for tasks 3 and 4 for repeatability testing (RQ2). P3 was asked to guess the glass and bowl sizes (RQ1) before performing the portion size task (RQ1). Sizes of bowls and glasses were randomized and counterbalanced. RL: real-life; L: large; M: medium; RQ: research question; S: small; VR: virtual reality.

In the virtual kitchen, participants were asked to wave at themselves (virtual avatar) in the mirror and explore how their movements matched the avatar before beginning with the study tasks. The participants then proceeded to grab the desired cereal box and pour it into the virtual bowl placed in front of them to the desired amount or to match with the previous RL condition. The participants then completed the task of pouring milk (plain or chocolate) into the virtual glass (refer P1 case in Figure 3). Figure 1B and C show the point of view of a participant for the bowl size selection task and cereal-serving task.

A subset of participants was asked to perform additional tasks related to RQ2 (repeatability) and RQ1 (container size estimation). For instance (refer to P2 case in Figure 3), within the same VR session, participants were first instructed to serve the food in RL and VR. Then they were instructed to serve again by imagining that they were serving breakfast for a subsequent day—with the goal of matching the exact portion of the food items as performed in steps 1 and 2. This task contributed data collection for RQ2 (repeatability). To avoid learning effects, they were presented with a different set of cereal and milk, and container sizes.

To answer RQ1 about container size estimation (refer P3 case in Figure 3), participants in the first condition were presented with a specific-sized bowl and glass. In the second condition (VR or RL), they were presented with 3 different sizes of bowls and glasses and were asked to identify the sizes they had seen in the first condition. “VR first” refers to the condition when participants were initially shown the bowl or glass in VR condition and then were asked to guess the container size in RL condition and vice versa for “RL first.” The sizes selected by participants were recorded. Regardless of their answer, the correct-sized bowl and glass were presented to them to help them complete the tasks for the second condition. In total, participants spent less than 5 minutes in VR and less than 10 minutes in total.

Data collection procedures varied across RQs to accommodate the high volume of museum visitors. While the P1 case was conducted during peak hours, the P2 and P3 cases (Figure 3) were restricted to off-peak periods (such as late afternoons) as they required additional time and physical setup. This scheduling was essential to manage the long queues for the popular VR exhibit and ensure a steady participant flow. Consequently, these variations in study design resulted in distinct participant pools for each RQ, as detailed in Table 2.

**Table 2.** Summary of data points for each research question (RQ).

Research questions and subtasks	Participants, n	Final data points after cleaning
RQ 1		
Food portion size	317	317 data points for cereal (both VR^a^ and RL^b^ conditions) 305 data points for milk (both VR and RL conditions)
Food healthiness	284	284 data points for cereal in RL condition 264 data points for cereal in VR condition 276 data points for milk in both VR and RL conditions
Container size	195	195 data points for bowl size guesses (72 RL first and 123 VR first) 193 data points for glass size guesses (70 RL first and 123 VR first)
RQ 2		
Presence, embodiment, and simulator sickness	411	411 total responses: 240 responses from age group of 5-8 years, and 171 responses from age group of 9‐12 years.
Repeatability	140	134 data points for cereal and 140 data points for milk—all data points were only in the VR conditions
RQ 3		
Demographics	420	180 male and 169 female participants; gender data not recorded for 71 participants 245 participants in age group of 5‐8 years; 175 participants in age group of 9‐12 years 195 data points for glass size estimation; 193 data points for bowl size estimation

a VR: virtual reality.

b RL: real-life.

### Methodological Challenges

Our study procedures were guided by the challenges of conducting a controlled study in a real-world setting with children. Challenges of testing prototypes in real-world settings and the need to adapt study design have been highlighted for field studies [64], which also guided our study design. First, we aimed at reducing the VR exposure time for children to manage potential simulation sickness. Hence, we developed short VR activities for children and simpler and shorter questionnaires for data collection.

Second, to ensure a smooth and safe experience in the busy science museum, we made a few adjustments to the study tasks for RL and VR conditions that were guided by the pilot testing. Unlike in VR, participants in the RL setting were not allowed to handle the food directly. This approach not only safeguarded our electronic equipment from food spillage but also aligns with how parents typically serve food to young children to avoid spillage. By having a researcher serve the food, we were also able to mitigate risks related to food allergies and hygiene.

Finally, due to the different affordances of virtual and real worlds, we used a mirror in VR as it is a common strategy [63] to induce ownership of a virtual body. Participants can connect to the virtual avatar by seeing their mirror reflection and associating their bodily movements with the avatar. However, in RL, it was not natural to see yourself in a mirror in a public setting (ie, amidst the crowd) when participants could directly see their body movements without any interruption. Hence, a mirror was not included in RL.

### Data Collection

**Figure 4.**
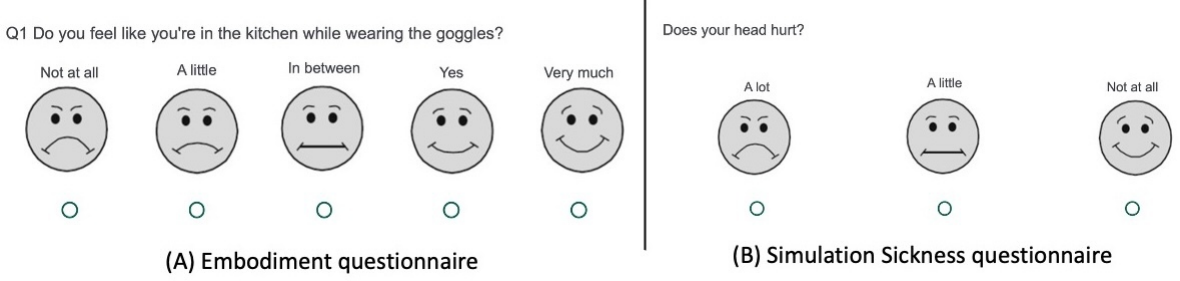
Snapshots of visual scales for (A) embodiment questionnaire and (B) Simulator Sickness questionnaire.

A researcher carried a tablet with Qualtrics questionnaires for data collection. Demographics information was noted based on the researcher’s observations of the participant's gender, skin tone, and hair color, which were used to match the VR avatar. Participants’ demographic details (age and gender) were collected from their parents during recruitment. After completing the study tasks, participants sat down with a researcher to complete a questionnaire with 17 questions structured in the following way: (1) 1 question on previous experience with VR; (2) 8 combined presence and embodiment questions, including 3 questions (Q1-Q3) about presence adopted from the iGroup Presence Questionnaire [65] and 5 questions (Q4-Q8) about embodiment adopted from Gonzalez-Franco [66]; (3) 7 questions on discomfort felt in completing the activity through an adopted sickness questionnaire [67,68]; and finally, (4) 1 question on subjective experience with VR. We combined the presence and embodiment questionnaires and reduced the number of questions to simplify data collection from our participants and to keep the study length manageable for children. The researcher read each question to the participants while showing the tablet with the visual Likert scales, as shown in Figure 4, and asked the participants to touch the desired option. The researcher also asked the participant to verbally explain their reasoning wherever relevant, which were noted down as a response to the last question. Researchers’ observations were also noted through a separate Qualtrics form.

Presence, embodiment, and sickness questions followed a visual scale as shown in Figure 4A. For the presence and embodiment questionnaire, the Likert scale was coded as not at all=1, a little=2, in between=3, yes=4, and very much=5, while the Likert scale for the Simulator Sickness questionnaire was coded as not at all=1, a little=2, and a lot=3. The Likert scale for presence and embodiment was reduced from a 7-point scale to a 5-point scale, while the sickness questionnaire was reduced from a 4-point scale to a 3-point scale. These changes were guided by our pilot testing with children, where children naturally used the descriptors—not at all, a little, and a lot—to respond to presence-, embodiment-, and sickness-related questions. These changes are aligned with a previous study [69], which emphasizes the importance of using simple and visual scales when designing questionnaires for children.

Study variables related to the virtual kitchen were recorded in the VR headset as a csv file. The csv files were transferred from the headset to a safe storage at the end of the day. The csv included data related to study condition (VR or RL), food type (healthy or unhealthy), given cereal type (Froot Loops and crunchy nuts as unhealthy cereals; Cheerios and cornflakes as healthy cereals), given milk type (chocolate milk as unhealthy option and plain milk as healthy option), and size of the given bowl or glass (small, medium, and large). These data also included the cereal weight (in g) poured in the virtual bowl, milk weight (in g) poured in the virtual glass, guess made by the participant about the container size (as small, medium, and large), and time taken to pour cereal or milk. For the RL condition, the researcher manually maintained an Excel sheet to record participants’ selection such as cereal weight poured in the bowl, milk weight poured in the glass, and guess made by the participant about the container size.

### Data Analysis

To answer our RQs, we conducted statistical tests using STATA (version 17; StataCorp LLC) on the data collected through the VR headset. We conducted chi-square tests, correlations, 2-tailed *t* tests, and kappa analysis to compare the level of agreement between study parameters. Kappa statistics [70] is more suitable for assessing agreement when multiple categorical factors are involved. Agreement cannot be measured through a chi-square test or a regression test, as they can only suggest correlation between the outcome and independent variables. All significant results are reported in the “Results” section. Descriptive analysis was conducted to analyze presence, embodiment, and simulator sickness collected through Qualtrics. Finally, we conducted deductive thematic analysis [71] on the subjective data collected through the researcher’s notes in Qualtrics, and participants’ responses on the last question in the poststudy questionnaire. Thematic analysis was conducted to mainly complement the findings gathered from the quantitative analysis, as per the mixed methods approach [72]. Initially, the first author (DA) created a separate Excel workbook with participants’ quotes and researchers’ notes, which had a total of 63 entries. She then coded the 63 entries deductively around the RQs, with code names as kitchen characteristics, avatar characteristics, presence, sickness, VR experience, VR issues, study tasks, and other issues. After finalizing the quantitative analysis, the first (DA) and second authors (TH) worked together to select the best representing quotes for the generated quantitative insights.

Table 2 presents a summary of the data points that guided all 4 RQs. Data were cleaned by excluding incomplete records and identifying outliers based on physical plausibility. Data points were discarded if the poured volume exceeded the maximum capacity of the assigned container (small, medium, or large). These logical bounds ensured that the final dataset included only realistic serving behaviors. To establish a baseline for outlier detection, the maximum capacity for each of the 3 container sizes (small, medium, and large) was determined by measuring the physical weight of the bowls and glasses when filled to capacity with cereal and milk. Consequently, the total number of valid data points (column 3 in Table 2) differs from the initial number of recruited participants. Because each child performed tasks in both VR and RL environments, they contributed multiple data points to the final dataset. In total, 437 participants contributed data to this study, with each participant contributing to 1 or multiple research data points. Hence, Table 2 distinguishes between the participant count and the valid data points used for analysis in each condition.

To address RQ1 related to food portion size, a paired 2-sample *t* test was conducted to find relations between the amount of food (milk or cereal) poured by the participants in VR and RL conditions. A total of 317 data points were used for cereal and 305 data points were used for milk.

To address RQ1 regarding food healthiness, we conducted a 2-sample *t* test assuming equal variances to compare the weight of unhealthy and healthy cereal and milk in both conditions. Healthy and unhealthy portion sizes were compared separately for RL and VR conditions. We had 284 data points for cereal in the RL condition, 264 data points for cereal in the VR condition, and 276 data points for milk in both VR and RL conditions.

In response to RQ1 regarding the container size, we analyzed how close participants’ guesses were to the correct container size. We first performed a weighted kappa analysis on the study conditions (VR or RL) and participants’ guesses to the container (bowl and glass) size. For weighted kappa analysis, we used weighted metrics for calculating agreement in a way that a perfect agreement was assigned a 100% weight, while the next closest option received a 75% score, and the third option received zero weight. We also conducted a chi-square test to supplement the results of weighted kappa analysis. There were 195 data points for bowl size guesses, and 193 data points for glass size guesses.

To analyze RQ2 regarding presence, embodiment, and sickness, we excluded incomplete responses, which led to a total of 411 responses. We analyzed these responses for 2 age groups: those aged 5‐8 years (240 responses) and 9‐12 years (171 responses). We split our analysis into 2 groups, as previous research suggests that children undergo different motor skill development at different ages [53,73,74], which may influence their interactions within VR.

The Likert responses were coded as numeric values from 1 to 5 for “Not at all” to “Very much,” and from 1 to 3 for “Not at all” to “A lot.” We calculated the mean of the responses for all questions under the same category. For instance, the average of Q1, Q2, and Q3 scores formulated the score for “Sense of Presence.” Finally, an embodiment score was calculated by taking an average of the responses for 3 categories: body ownership, self-location, and control.

To investigate the repeatability of tasks in VR (RQ2), we had 134 data points for cereal and 140 data points for milk. A paired 2-sample *t* test was performed to compare the amount of cereal and milk poured by participants in VR.

Finally, for RQ3 regarding the effect of demographics, we again conducted kappa analysis to find the influence of age and gender on the amount of food (milk or cereal) served in VR and RL conditions, as well as in guessing the container sizes. The analysis was done separately for boys and girls, younger than and older than 8 years, that is, aged 5‐8 and 9‐12 years. There were 306 data points for cereal and 295 data points for milk.

## Results

We structure our findings around the 3 RQs. A total of 437 children participated in our VR study. Out of 437 participants, 328 children had no experience with VR and 109 children had used VR at least once.

### RQ1: Food Portion Size (Being Served in RL Versus Serving Themselves in VR)

As per the paired 2-sample *t* test, the mean value of milk poured in VR is 242.5 (SD 8623.6) g and in RL is 215.9 (SD 9073.9) g. For both conditions, the SDs for the mean value of milk poured are high. The high SDs are due to the high variability of milk content poured by the participants. The mean value of milk poured in VR was significantly higher than in RL (*t*_304_*=*3.87; *P*<.001).

We also conducted the paired 2-sample *t* test to understand the order effects (VR first vs RL first) on milk portion sizes. There was a significant difference for participants who completed VR first (*t*_147_=6.37; *P*<.001). Children poured more milk in VR (mean 258.9, SD 9153.2 g) than in RL (mean 212.5, SD 9827 g) when they completed the VR condition first. However, if the participants completed the RL condition first, there was no significant difference in milk weight between the 2 conditions.

We conducted a paired 2-sample *t* test, which shows that the mean value of cereal weight in VR (mean 54.1, SD 796.3 g) was significantly higher than the mean value of cereal weight in RL (mean 41, SD 388.6 g; *t*_316_=8.16; *P*<.001). This shows that children served themselves more cereal in VR than what they were served by the researcher in RL. The SD for the mean value of cereal poured is much higher in the VR condition than in RL.

We conducted the paired 2-sample *t* test to understand the order effects (VR first vs RL first) for cereal. Regardless of which condition was completed first, there was a significant difference that participants served more cereal in VR than in RL. For participants who completed the VR condition first, cereal weight in VR (mean 57.2, SD 826.7 g) was significantly higher than in RL (mean 43, SD 454.7 g; *t*_147_=5.71; *P*<.001). For participants who completed the RL condition first, cereal weight in VR (mean 51.3, SD 758 g) was significantly higher than in RL (mean 39.1, SD 323.7 g; *t*_165_=5.83; *P*<.001).

### RQ1: Food Healthiness

As per a 2-sample *t* test, the mean value of unhealthy cereal poured in RL (mean 48.4, SD 461.1 g) was significantly higher than the mean value of healthy cereal weight in RL (mean 34.2, SD 258.8 g; *t*_284_*=*6.31; *P*<.001). This shows that participants served themselves more portions of unhealthy cereal in RL than the healthy cereals. Similarly, the mean value of unhealthy cereal poured in VR (mean 63.1, SD 934.1 g) was significantly higher than the mean value of healthy cereal poured in VR (mean 41.1, SD 225.2 g; *t*_264_=−7.47; *P*<.001). This shows that participants served themselves more portions of unhealthy cereals in VR than the healthy cereals. High values of SD are due to the large variability of the food selection made by children. The healthiness of the milk had no significant effect on the serving sizes chosen by the participants in either the VR or RL conditions. These findings confirm that children’s attitude toward food healthiness is similar in both VR and RL.

### RQ1: Container Sizes

The weighted kappa statistics showed no agreement (κ=0.34) between the given and guessed size of bowls between VR and RL conditions. This shows that children cannot accurately match the size of the serving bowl between VR and RL. Participants made more incorrect guesses in VR first than in RL first, with a correct to incorrect distribution ratio of 59:64 and 46:26, respectively, as shown in Table 3. Chi-square test showed significant differences for small-sized bowls (*χ*^2^_1, 53_=12.0; *P*=.001). This suggests that children cannot match the small-sized containers in RL correctly if the bowl is first shown in VR. No significant differences were found for medium-sized and large-sized bowls. These results suggest that children are better at matching the medium-sized containers, regardless of whether they saw them in VR or RL first.

**Table 3.** Guesses for bowl and glass based on conditions. VR^a^ first means that participants were shown the bowl or glass in the VR condition first and then were asked to guess the size in the RL^b^ condition.

Conditions	Small (correct/incorrect)	Medium (correct/incorrect)	Large (correct/incorrect)	Total (correct/incorrect)
Bowl VR first	9/29	37/9	13/26	59/64
Bowl RL first	12/3	21/10	13/13	46/26
Glass VR first	10/32	17/26	20/18	47/76
Glass RL first	11/10	19/9	13/8	43/27

a VR: virtual reality.

b RL: real-life.

For glass size guesses, we similarly performed weighted kappa analysis and found no agreements (κ=0.20) between the given and guessed size for the VR and RL conditions. This shows that children cannot accurately match the size of the serving glass between VR and RL. Similar to the bowl size condition, participants made more errors in guessing glass sizes in the VR-first condition than in the RL-first condition, with distribution between correct and incorrect guesses as 47:76 and 43:27, respectively. Chi-square test showed nonsignificant differences for all sizes of the glass. The results (correct-to-incorrect ratio for different sizes) in Table 3 suggest that children are better at matching different-sized glasses in VR after first seeing them in RL.

### RQ2: Presence, Embodiment, and Simulator Sickness

Table 4 presents the presence and embodiment scores for 2 age groups: those aged 5‐8 years (n=240) and 9‐12 years (n=171). The overall scores for each factor are similar for both groups. The mean embodiment score of 3.1 suggests that participants felt a moderate or ambiguous sense of ownership and control regarding their virtual body and its location in VR. Similarly, Presence received the mean score value of 2.9 for both groups, suggesting that participants partially felt that they were present in VR. On the other hand, control received a relatively higher mean score for both groups: 3.5 for those aged 5‐8 years and 3.6 for those aged 9‐12 years.

We also analyzed the simulator sickness score for the 2 age groups: those aged 5‐8 years and 9‐12 years and found similar results. Table 5 presents a cumulative mean score of 1.10 (SD 0.32) and 1.12 (SD 0.33) for younger and older children, respectively. The low sickness scores suggest that participants did not feel any simulation sickness in VR. Some participants, however, mentioned feeling eye fatigue after the study.

**Table 4.** Presence (denoted as P) and embodiment (denoted as E) scores on the Likert scale (1 being “Not at all“ to 5 being “Very much”) for age groups of 5‐8 and 9‐12 years.

Factors	Questions	Age group (5‐8 years), mean (SD)	Age group (9‐12 years), mean (SD)
P: Presence	Q1. Do you feel like you’re in a kitchen while wearing the goggles? Q2: Do you feel like you’re still in the museum? Q3: Does the kitchen feel real?	2.9 (1.1)	2.9 (1.1)
E: Body ownership	Q4: Does the virtual hand feel like your hand? Q5: Does the virtual body feel like your body?	2.6 (1.4)	2.5 (1.2)
E: Self-location	Q6: Do the virtual hands appear where your hands are? Q7: Does the virtual body appear where your body is?	3.2 (1.1)	3.4 (1.0)
E: Control	Q8: Do the virtual hands move like you want them to?	3.5 (1.0)	3.6 (0.95)
Embodiment score (Inclusive of Q4-Q8)		3.1 (1.2)	3.2 (1.0)

**Table 5.** Sickness score on the Likert scale (1 being “Not at all” to 3 being “A lot”) for the age group of 5‐8 and 9‐12 years.

Question ID	Question	Age group (5‐8 years), mean (SD)	Age group (9‐12 years), mean (SD)
S1	Do you feel sick?	1.1 (0.4)	1.1 (0.2)
S2	Does your head hurt?	1.1 (0.4)	1.1 (0.3)
S3	Do your eyes hurt?	1.1 (0.3)	1.2 (0.4)
S4	Is your stomach upset?	1.1 (0.3)	1.1 (0.3)
S5	Are you dizzy with your eyes open?	1.2 (0.4)	1.2 (0.4)
S6	Are you dizzy with your eyes closed?	1.1 (0.3)	1.1 (0.3)
S7	Do you feel like burping at all?	1.1 (0.2)	1.1 (0.4)
Sickness score (Inclusive of S1-S7)		1.1 (0.3)	1.1 (0.3)

### RQ2: Repeatability

A paired 2-sample *t* test did not show any significant difference between the first and second trials for pouring milk (*t*_139_*=*0.19; *P*=.84) and cereals (*t*_133_*=*0.77; *P*=.44). These values could suggest that VR offers a reliable test-retest mechanism for food and drink with respect to portion size.

### RQ3: Demographics

Kappa statistics were used to assess the level of agreement between VR and RL container size estimations across different demographic subgroups. The results indicated no significant agreement between the 2 conditions when analyzed by age or gender (see Table 6 for details). Similarly, no significant agreement was found between VR and RL food portion sizes across these age and gender groups.

**Table 6.** Summary of kappa statistics for container size estimation.^a^

	κ values for glass size estimation	κ values for bowl size estimation
Male	0.15 (n=108)	0.39 (n=107)
Female	0.28 (n=87)	0.26 (n=86)
5‐ to 8-year-olds	0.18 (n=120)	0.33 (n=118)
9‐ to 12-year-olds	0.24 (n=75)	0.35 (n=75)

a All κ values are less than 0.40 and hence considered small.

### Qualitative Results

To better understand the quantitative findings presented in the previous section, now we present the qualitative findings that were generated from participants’ responses and researchers’ notes.

### Higher Food Portion Sizes in VR

We found multiple reasons for higher food portion sizes in VR than in RL (RQ1). First, there is no sense of weight in VR, such as the weight of a milk can or serving bowl. The lack of weight in VR led to a “mismatch” in motor expectations. For instance, when lifting the virtual 2-liter milk can, participants often overcompensated for its expected weight, leading to exaggerated movements and accidental spilling. We included any food that landed in the bowl or glass in our final measurements because these spills were not technical errors or outliers; rather, they were a fundamental result of the participant’s interactions with the VR interface. Removing them would have artificially inflated the accuracy of the VR condition.

Second, the VR food environment was found fun by participants, where they were intrigued by the novelty of the VR and/or the task presented. Participants turned the simple task of pouring food into a playful activity, where they continued pouring to overflow the containers. This is evident by high SD values for cereal and milk portion sizes in VR as compared with the RL condition (as discussed under section “RQ1: Food Portion Size”). As such, children enjoyed the freedom to interact with food in VR and tried different activities to test the virtual environment (eg, throwing containers, turning containers over to check gravity, etc). Although participants received identical instructions to serve themselves food in both conditions (VR and RL), significant variations in portion size were observed. These discrepancies likely reflect differences in how the younger cohort interpreted or retained instructions across environments.

Finally, since the children did not handle real food in RL, this pseudopouring could have created a difference. For example, participants might not want to be judged by the researcher on their intake and hence agreed on smaller portions of food in RL. Interestingly, we identified an *order effect* specifically for milk portions: participants who completed the RL condition first were more likely to replicate similar volumes in VR. This suggests that the visual transparency of the RL glassware provided a sensory anchor that guided subsequent actions in VR. In contrast, when the VR condition was performed first, the lack of haptic weight, the playful nature of the environment, and different perception of size in VR [75] might have led to significantly larger milk portions that did not align with subsequent RL servings. The RL bowl, on the other hand, was opaque; hence, the visual guidance was limited to influence participants’ behaviors in VR.

### Lower Presence and Embodiment in VR

In our study, presence in the VR experience and embodiment of the VR avatar received lower scores from the participants (RQ2). In the poststudy questionnaire, many participants commented that the virtual kitchen did not resemble the kitchen at their home. As said by one of the participants, “It’s not my own kitchen!.” Another mentioned, “The table wasn’t like a kitchen table.” Participants looked at how realistic the kitchen and the surroundings were, and the limitations with real-world resemblance reduced their sense of presence in the virtual kitchen. The participants strongly felt that they were in the museum while doing the VR activity. The concept of the virtual world was new to them, and they could not imagine being at 2 places—both in the real and virtual worlds simultaneously.

The low score for the control factor for 5- to 8-year-olds is in line with our observations, as younger children could not use the controllers effectively. They found it challenging to navigate the VR tasks through controller buttons and movements. On the other hand, presence received the same scores from both age groups, and self-location was found higher in older children than in the younger age group (5-8 years).

Low scores for body ownership suggest that participants did not feel embodied with the VR avatar. Participants provided comments on avatar resemblance, physicality, and fidelity. One participant mentioned in the poststudy questionnaire, “Hands do not feel real because real hands can’t go through the table.*”* Another participant commented on the nonhuman characteristics of the avatar: “The boy [avatar] did not blink. It looked like a ghost.” One participant noted in the questionnaire: “The body feels like a cartoon. You can’t feel the hands; you can’t move the fingers.” Participants were looking for sensory sensations in their hands while doing actions in VR, and perfect matching of the virtual avatar with their body characteristics (eg, skin color, hand size, body size, etc)—the absence of which reduced their ownership of the virtual body.

## Discussion

### Principal Findings

Our study investigated the potential of using VR to conduct food choice studies with children. We examined their perception of food characteristics (portion size and container size) and their experience of being in VR (embodiment, presence, and repeatability) and compared them with the RL interactions at a museum. We found key differences in terms of food portion sizes (RQ1) as children served themselves more food in VR than in RL settings, along with some similarities as children served themselves more portions of unhealthy food in both VR and RL. For container size mapping (RQ1), we found that children were good at matching the medium-sized bowls more accurately, regardless of whether they saw them in VR or RL first, and they are better at matching different-sized glasses in VR after first seeing them in RL. On the other hand, presence and embodiment scores (RQ2) were found low, whereas control was higher for both age groups of 5‐8 and 9‐12 years. Additionally, VR qualified the test-retest mechanism (RQ2) for both food and drink, as children were able to repeat the same tasks in VR. Finally, we found no influence of demographics on container size estimation or food portion size (RQ3).

Our study revealed that the effectiveness of a virtual food environment is influenced by factors related to both VR’s affordances and the study’s setting. These factors include the differences in handling food in VR and RL conditions, level of engagement in VR versus RL, and the limitations of VR in supporting tactile and sensorial interactions. These differences highlight how VR, as a novel interaction medium, offers a distinct experience from RL surroundings, due to which a direct mimicry of RL interactions is neither feasible nor should be expected. The study also highlighted challenges related to ensuring data accuracy with VR and inquiring about the real and virtual worlds in the questionnaire. Consequently, our study suggests that the current form of VR is not suitable for conducting food choice studies with children. Next, we discuss our findings into 2 themes and present 4 design recommendations to guide effective VR experiences for this research context in future.

### Playfulness Versus Science

#### Overview

Our results showed that children served themselves more food (both cereals and milk) in VR than in RL (RQ1). The SD was also much higher for VR than for RL, likely because children found the VR environment engaging and playful, wherein they dismissed the study instructions to explore the VR space (such as testing how food would overflow). Conversely, in RL, children were assisted by a researcher during the pouring tasks, which limited their opportunities for play. Despite the playful behavior in VR, we found that participants in both conditions served themselves more unhealthy food. This suggests that the differences in food-handling methods did not influence the children’s overall food choices.

While extending the familiarization phase to mitigate the novelty effect may seem beneficial, it presents significant risks regarding participant fatigue and disengagement, particularly within the 5‐12 years age demographic. Prolonged exposure to VR environments can be cognitively taxing for younger children. Consequently, we used a brief orientation to ensure that participants were proficient with the mechanics while maintaining the focus required for reliable data collection. This approach allowed us to capture more authentic behavior before the onset of boredom or cognitive exhaustion.

Prior work has shown that VR can put children in a positive mindset, even during uncomfortable medical therapy [32]. Another study [76] explored the balance between playfulness and learning, in which playfulness is a conceptualization of intrinsic motivation, suspension of reality, and internal locus of control. In our study, the lack of consequences for actions in VR amplified 2 of these aspects of playfulness: suspension of reality and internal locus of control. Children enjoyed the VR environment and the freedom of controlling their food serving without supervision increased their excitement. Research on ethical practices of involving children with VR [77] highlighted that children are easily excitable within VR worlds; hence, their exposure should be limited. We observed this effect firsthand, although our virtual environment was a kitchen and not designed to be explicitly playful. The excitement for the VR experiences was also evident by the fact that our study station was the most popular among all other stations in the study room and still children were happy to wait for their turn.

While playfulness increased the likability of VR among children, it raised challenges for using it as a scientific tool. Engagement came at the cost of accuracy and precision, diminishing the quality of the data we collected on food selection. These insights raise important questions about how to use VR for scientific studies with children, for which we provide 2 guidelines next.

#### Recommendation 1: Design Supervised VR Food Experiences

To achieve greater validity in food choice studies with children, we suggest developing supervised VR food experiences that include a moderator in the virtual environment. The moderator, who could be a member of the research team, could guide children through the required activities such as selecting food or containers. While some attempts have been made to develop supervised VR experiences, for example, to encourage equal participation in moderated online meetings [78], engaging children in supervised VR experiences for scientific activities has not received any attention yet.

The role of the moderator and their interactions within the environment must be carefully considered, as they should not overshadow the children’s interactions in the virtual food environment. Additionally, it is also important to explore how a moderator could be embodied as an avatar (eg, humanoid or nonhumanoid) to encourage greater engagement from children of different ages. Having a moderator might lead to altered behavior, which aligns with the existing literature on the Hawthorne effect [79,80]. However, as per Slater [81], there are always a multitude of variables that could potentially influence the results, and he recommended using data triangulation—which could be employed when designing and testing supervised VR food experiences for children.

#### Recommendation 2: Design Gamified VR Food Experiences for Children

Building on the insights from our study that VR was a fun experience for children, we suggest developing gamified VR experiences. Recent research has used gamified VR to prepare adolescents (aged 10‐16 years) for the rigors of magnetic resonance imaging scans [82]. By engaging in a “statue game” with a virtual panda, children practiced physical movements that mirrored the scan procedure, such as reclining on a bed in synchronization with the virtual character. This study demonstrated that interactive VR environments, combined with real-time feedback, effectively increase task compliance and minimize the involuntary motion that often compromises medical imaging. Gamified VR experiences could support children’s intrinsic motivation to find play in everyday activities [76,83]. However, such experiences must be carefully designed so that the game elements encourage children to perform the required tasks more accurately and discourage behaviors that go against the study goals. Points and award systems can be used to positively reinforce children within the game. For instance, players could receive points based on how cleanly (ie, without any spillage) they perform a pouring task in VR. Consequently, gamification would not only increase children’s willingness to perform the task but also enable researchers to use the virtual medium more appropriately.

### “It’s Not My Own Kitchen!”: Presence and Embodiment

#### Overview

Our study revealed that children have a high threshold for realism with both their virtual avatars and the VR environment. The lack of realism created issues, as children struggled to embody the virtual avatar and did not feel present in the virtual kitchen (RQ2, Table 4). The participants’ behavior in our study is typical of the Goldilocks problem [84]. In the popular children’s story, Goldilocks seeks a chair and porridge that are “just right”—not too big or too small, not too hot or too cold. Similarly, our participants desired a virtual body avatar and kitchen setting that felt “just right” to them. This is an age-appropriate behavior, as children (unlike adults) engage in an internal dialogue to identify objects based on their prior knowledge (ibid.).

For embodiment, Slater [81] explained the differences in embodiment levels across age groups by looking at the biases we develop as we get older. The authors argued that as adults, our concept of “real” in VR is biased. Since adults already know the limitations of the technology, we are more likely to accept certain nonrealistic aspects of a virtual world as being “realistic” enough. Likewise, adults understand that the behavior of virtual objects may deviate from the real world, and they would still find it an acceptable interactive experience.

Our findings are in line with prior works [52,54,55], as they all highlight the importance of human-like avatars for children to feel embodied in VR. For example, Liao and colleagues [55] also reported low embodiment in a study with 6‐ to 8-year-old children, as the VR experience was described as not real. However, physiological and emotional responses collected during that study suggested the children were immersed in the virtual environment. Research on the cognitive development of children [85] suggests that younger children decipher between “real” and “unreal” based on their prior perception and reasoning of their surrounding world. Our study confirmed this observation, as participants still found the VR experience very engaging despite providing low presence scores. Further studies are required to understand if and how low embodiment in VR influences the food portion sizes children serve.

In our study, control was found to be slightly higher for older children (aged 9‐12 years) with a mean score of 3.6 (SD 0.95) as compared with the younger group (aged 5‐8 years) with a mean score of 3.5 (SD 1.0)*.* Control is correlated with their sense of self-location, and older children therefore also had a better sense of self-location than younger children. We found that younger children found it challenging to maneuver the VR controllers for the required tasks, whereas older children demonstrated better motor skills. We also observed that younger children were losing their balance while performing the task of pouring and tilting in VR (it is important to note that no accidents were reported, as children were seated and were in constant supervision). These findings are consistent with prior literature on movement skill development [86], which suggests that control skills are still developing in children aged 1‐7 years, and therefore they require greater coordination to perform such tasks.

Presence was also found low in our study, potentially because of the language used in the instructions. By asking children to imagine being in their own kitchen and serving breakfast (as described under section “Study Procedure”), we unintentionally led them to compare the virtual environment with their actual home kitchen in terms of layout and food items. Moreover, participants also commented that the virtual hands did not feel real, thus contributing to low scores. Despite the low scores, children enjoyed the overall activity.

These insights raise important questions that require further investigation. For instance, is it possible for children to enjoy VR activities without being “present” in the virtual world? How important is high embodiment for children in VR? Finally, how can we design efficient tools to measure children’s presence in VR? Next, we discuss 2 guidelines on how to enhance children’s immersion in VR.

#### Recommendation 3: Design Virtual Food Selection Tasks Around Children’s Motor and Perceptual Abilities

In our study, the VR experience primarily involved 2 interactions: grabbing an object (a cereal box and milk can) and tilting it onto a bowl or glass, respectively. We found that younger children (aged 5‐8 years) struggled with these tasks because they required complex control through the VR controllers. Owing to the developing motor skills in children of various age groups, we suggest that designers should carefully develop the virtual tasks around the motor capabilities of the target age group. Additionally, designers should not try to exactly mimic the real-world interactions with food in VR as they may not be feasible. For instance, the pouring task in our study was problematic, as there is reduced weight perception in VR, which led to accidental spillage. Hence, we suggest mapping the real-world tasks to simpler actions within VR such as grabbing or moving.

Our study also indicated that children are more likely to perceive food container sizes correctly for the “medium” size (RQ1). As per Piaget’s discussion [87], children younger than 11 years are known to have difficulty in estimating the object volume. For instance, such children focus only on 1 dimension of an object and tend to ignore others. Hence, they find it difficult to understand that a quantity does not change if it has been altered (eg, by stretching, shrinking, spread out, etc). Similarly, another study [75] suggests that adult participants perceived sizes very differently between VR and RL, and that in VR, they were more reliant on the familiar sizes to perceive the size of unfamiliar objects. In our study, when we asked children to pick the same bowl in VR as they had picked in RL and vice versa, children could make correct estimations only for medium size. They were able to identify all 3 sizes of glasses in VR if they had seen them first in RL. With the VR first condition, more incorrect guesses were observed. Our study suggests that apart from the cognitive inabilities and abilities of our target group, we anticipate that the virtual environment adds another layer of complexity that makes the size estimation challenging for children.

Participants guessing the “medium” size containers more accurately than other sizes could also be understood through the Goldilocks problem [84], where children selected “just the right kind” of containers for themselves. For instance, children aged 5‐12 years are typically served in small- to medium-sized containers at home to prevent food wastage. Participants may have selected medium-sized containers due to their everyday exposure to that size. Moreover, previous works in object size perception [50,88] also indicate that object sizes in VR have an inverse correlation with virtual hand sizes, under all degrees of avatar realism. In our study, we used a single size avatar for all participants, without scaling to participants’ bodies. Hence, further studies are required to understand the connection between avatar size and container size selection for children.

Our findings related to container sizes also suggest that it is more efficient for future studies to have a single size. Additionally, it was challenging to replicate the continuous variation in food amount while being poured into virtual containers in VR. Therefore, for future studies on food selection in VR, we suggest that researchers should focus on using discrete portion sizes, such as different slices of pizzas or a bowl of soup instead of involving actions such as pouring or serving food through spoons.

#### Recommendation 4: Design Visual Embodiment and Presence Questionnaires for Children

Our final recommendation relates to how to measure children’s embodiment and presence. In our study, we found that children were unable to understand the questions related to the real and virtual world. For instance, questions related to embodying an avatar were difficult for children to interpret, as the virtual body and hands were neither their own nor very realistic. As such, children were very focused on their RL experiences and were unable to connect to an unfamiliar kitchen and unknown avatar. Our concerns with the existing questionnaires resonate with the concerns raised by other researchers [49,89], as they also noted the methodological challenges of using questionnaires and emphasized the importance of using other measures to better understand participants’ perceptions of the virtual world.

Slater [81] argued that presence is not a natural phenomenon, as we do not think about it in our everyday life. Also, unlike other feelings such as anxiety, presence cannot be easily compared with our previous life experiences. Since children have fewer RL experiences than adults, their familiarity with different concepts in VR may differ. Hence, researchers should inquire about embodiment on parameters that children can easily compare with. Additionally, presence should be inquired against familiarity. For instance, questions related to familiarity with the designed experience should be asked to understand children’s perspective. Designers should also build VR experiences that children are likely to be familiar with to help them develop valid connections in the virtual environment.

New tools are, therefore, required to inquire about measures of embodiment and presence of children in VR. Prior work [90] developed a visual questionnaire to understand the perceived physical literacy of children. The questionnaire shows a “bunny” character performing different physical activities. Children interpret the bunny’s activities and indicate their literacy level around physical activities. Using an animal-based visual questionnaire helped children to focus on the question being asked without focusing on the finer details of the bunny character. Similarly, using animal avatars with children in VR has also received positive responses in previous research as realistic behaviors were not expected from animal characters [54]. Visual questionnaires coupled with animal characters thus present a promising direction toward measuring children’s embodiment and presence within VR.

### Limitations

This study has certain limitations, which might influence the generalizability of the study findings. First, there was a difference in how the VR and RL tasks were managed in our study. In the VR condition, children poured their own food, but in the RL condition, they did not. This was a necessary limitation due to the study’s setting in a public museum, where the risk of spillage could impact other visitors and damage the VR equipment. Although we anticipated that the food portion sizes would be similar between both conditions, they varied. We found that this variation was not only due to the difference in pouring mechanisms but also because the novelty of VR combined with a lack of supervision within VR—which led children to treat it more as a playful activity. This highlights why our recommendation to design supervised experiences is an important consideration for future studies.

Second, children were not allowed to eat the food they served themselves due to food safety reasons such as allergies. Since the amount of food served may differ from the amount consumed, further research is needed to understand the relationship between these 2 variables. Moreover, we used an average avatar size and did not adjust the VR kitchen or its features such as container sizes to account for each child’s hand size or age, which may have influenced our findings. Moreover, since eating practices vary across cultures, some may not consider cereals as a healthy option. While it is infeasible to choose a food item that satisfies every culture, our choice of food items was driven by the health star rating and sugar content mentioned on the package.

Third, due to the complexity of multiple tasks for each RQ, as well as the context of conducting research in an active public setting of a popular museum with multiple data collection stations, data loss across different tasks was an issue, causing imbalance of participant numbers across each RQ. Additionally, the playful behaviors of the participants of continuously pouring food in VR caused significant data loss for VR conditions.

Another limitation of our study is that it was conducted with an age group younger than the VR industry’s recommended demographic. Although we received ethics approval and implemented safety protocols, such as limiting VR exposure and ensuring that children remained seated, the design of the headsets may have influenced the children’s experience and our findings. The implication of the study could be limited to the specific hardware setup that was used, which was the Oculus Quest 2. Moreover, our participant sample may not be representative of all socioeconomic groups. Because the study was conducted at a museum with an entry fee for accompanying adults and free access to children, the findings may not generalize to children from other socioeconomic backgrounds.

Finally, we acknowledge that the modified embodiment and simulator sickness questionnaires may have impacted the results. These changes were necessary to simplify the questions and make them suitable for child participants.

### Conclusions

This paper presents insights from a controlled study conducted with 437 children (aged 5‐12 years) at a science museum. We investigated the potential of using VR as a tool for food choice research by measuring how children perceive food portion size and container size, and how their experience in VR compares with an RL setting. Our study raised important questions about how to collect accurate data with children using VR, how to design immersive experiences for them, and how to create a valid questionnaire to measure their experience in VR. In response, we present 4 recommendations around designing supervised virtual food experiences, applying gamification to ensure data accuracy, developing virtual tasks around children’s motor abilities, and designing visual questionnaires to inquire about children’s immersion in VR. Despite certain limitations, our study presents the first understanding of how children perceive food in VR, how they behave in a simulated food environment, and finally how VR could serve as a potential measuring tool for measuring their eating behaviors. These insights offer foundational knowledge for future VR research with children in the domains of HCI and health, while also opening new avenues for researchers at the intersection of VR and food science.

## Abbreviations

HCI: human-computer interaction
RL: real-life
RQ: research question
VR: virtual reality
